# Metformin Monotherapy With and Without Lifestyle Changes Affects Anthropometric Parameters, Blood Pressure, Blood Glucose, and Lipid Profile in Indian Patients With Newly Diagnosed Type 2 Diabetes

**DOI:** 10.7759/cureus.62131

**Published:** 2024-06-11

**Authors:** Aniruddha Sen, Amar Preet Kaur, Indu Saxena

**Affiliations:** 1 Biochemistry, All India Institute of Medical Sciences, Gorakhpur, Gorakhpur, IND

**Keywords:** fasting blood glucose (fbg), glycated hemoglobin (hba1c), diabetes mellitus type 2, metformin monotherapy, lipid profile, lifestyle changes, blood pressure, anthropometric parameters

## Abstract

Introduction: Type 2 diabetes mellitus (T2DM) is a consequence of insulin resistance, insulin deficiency, or both. It is usually seen in adults and is a consequence of genetic (polygenic inheritance), endogenous (obesity and or hormonal factors), and environmental factors (e.g., obesogenic environment, endocrine disrupting chemicals, stress, and medicines). The prevalence of T2DM has increased over the past few decades. South Asians, including Indians, are more prone to central adiposity and develop lifestyle diseases like T2DM at body mass index values lower than those considered normal for the Western population. Generally, the first line of treatment is metformin monotherapy with lifestyle changes in patients with T2DM. Most of the research conducted on this drug is on Western subjects. Since the Indian population has genetic differences in the site of deposition of adipose and is more prone to develop lifestyle diseases, the effect of metformin may be different in Indians.

Methods: Seventy-one (34 female, non-pregnant, non-lactating) adults with newly diagnosed T2DM were recruited in this short-duration pilot study after obtaining written informed consent. Patients regularly taking any drug were excluded, as were patients with chronic comorbidities. Treatment was initiated with metformin 500 mg OD. Lifestyle changes were recommended according to the age and physical condition of the patients. Anthropometric parameters (age, weight, height, BMI, waist-to-hip ratio (WHR), and waist-to-height ratio (WHtR)), blood pressure, glycemic status (fasting and 2 h PP glucose and HbA1c), and lipid profile of the subjects were recorded before initiating and six months after initiating metformin monotherapy with lifestyle changes.

Results: Small but statistically significant improvements were observed in the WHR,WHtR, blood pressure, blood glucose, and glycated hemoglobin. Although improvement was also observed in weight and lipid profile, these changes were not statistically significant.

Conclusion: This study shows that metformin monotherapy with lifestyle changes is suitable for patients of Indian origin and results in improvement in the WHR, WHtR, blood pressure, plasma glucose, and glycated hemoglobin.

## Introduction

Diabetes mellitus (DM) is a heterogeneous condition characterized by hyperglycemia and metabolic dysregulation. It can be of different types, depending on the underlying cause [1]. Type 2 diabetes mellitus (T2DM) is a consequence of insulin resistance, insulin deficiency, or both. The prevalence of DM has increased over the past few decades. It is now a major non-communicable disease in the developing countries of the world, including India, and has serious consequences on the economic condition of the affected family and the nation [2]. With almost 77 million adults with T2DM, 25 million adults with prediabetes reside in India, and more than 50% of people are unaware of their diabetic condition [3]. South Asians, including Indians, are more prone to central adiposity and develop lifestyle diseases like metabolic syndrome, T2DM, hypertension, fatty liver disease, and heart diseases at body mass index (BMI) values lower than those considered normal for the Western population [4,5]. Metformin (a relatively cheap and safe biguanide) is the most frequently initiated medication, prescribed as the first drug to more than 80% T2DM patients [6,7], along with lifestyle modifications. Metformin decreases the hepatic production of glucose, increases the extrahepatic utilization of glucose, increases fatty acid oxidation in skeletal muscles, decreases systemic inflammation, and increases insulin sensitivity [8]. Metformin has been reported to cause weight loss [9] and improve hyperglycemia [10] and lipid profile [11] in patients with T2DM. It has also been shown to have cardioprotective effects [12]. Since the deposition of adipose is different in persons of Indian origin and insulin resistance occurs at lower BMI values than in Caucasians [4,5], it is possible that the effect of metformin may also be different in magnitude.

This pilot study compares the effect of metformin alone and in combination with lifestyle changes in Indian patients with newly diagnosed T2DM. We have been unable to find original research articles in which the effects of metformin monotherapy on hyperglycemia, lipid profile, anthropometric parameters, and blood pressure have been studied in the same group of subjects of Indian origin, with newly diagnosed T2DM.

## Materials and methods

This interventional pilot study was initiated after obtaining ethical permission from the Institutional Human Ethics Committee (reference no. IHEC/AIIMS-GKP/BMR/119/2023). A convenience sample of newly diagnosed diabetic patients visiting the outpatient section of the Medicine Department of All India Institute of Medical Sciences, Gorakhpura, a tertiary academic hospital in Gorakhpur, India, was recruited in the study. Seventy-one patients (age 30-50 years) were recruited in the study after obtaining signed informed consent. The inclusion criteria included patients with newly diagnosed T2DM who had been advised lifestyle changes and initiated on 500 mg OD metformin monotherapy, non-smokers, and teetotalers. The exclusion criteria included pregnancy, lactation, and the presence of any other chronic illness (e.g., cardiovascular disease (CVD), hypertension, hormonal imbalance, nonalcoholic fatty liver disease (NAFLD), and cancer) besides T2DM.

In addition to metformin monotherapy, the participants were advised to implement lifestyle modifications (dietary changes and regular exercise) according to the American Diabetes Association (ADA) guidelines. 

All participants were interviewed at the time of recruitment regarding their dietary habits and physical activity level. They were requested to report all dietary changes and physical activities adhered to during the duration of the study, and the reasons for the inability following lifestyle interventions. After six months of treatment, the participants were also asked to report the side effects of the treatment and any other problems encountered related to the treatment.

The age, gender, weight, height, and circumference of the waist and hips of all the participants were recorded at the time of recruitment and after six months of prescribed treatment. The body mass index (BMI), waist-to-hip ratio (WHR), and waist-to-height ratio (WHtR) were calculated. Resting blood pressure and heart rate were recorded at the time of recruitment. 

The assessment of blood glucose and lipid profile was done at the time of recruitment and again after six months of treatment. Fasting venous blood samples were used for the measurement of fasting glucose, HbA1c, total cholesterol, HDL-cholesterol, and triglycerides. Two-hour postprandial samples of venous blood were obtained for measuring glucose.

Estimation kits from Erba Mannheim (Mannheim, Germany) and a fully automated analyzer from Transasia Bio-Medicals Ltd. (Mumbai, India), model XL 1000, were used for the measurement of glucose, glycated hemoglobin, and lipid profile. The procedure provided with each estimation kit was followed. LDL-cholesterol was calculated using the Friedewald formula [13]: [Total Cholesterol] = [HDL-cholesterol] + [LDL-cholesterol] + [Triacylglycerol]/5.

Data obtained were compared between genders and before and after treatment by using a T-test.

## Results

A total of 71 subjects (34 female) were enrolled in this study. All subjects continued to participate in the study for the six-month duration of this study. Although all the male subjects reported strict adherence to metformin monotherapy and lifestyle changes (regulation of diet and regular exercise prescribed according to the patient’s physical condition), only three female subjects reported adherence to lifestyle changes. Table 1 compares the anthropometric parameters between female and male subjects at the time of initiation of the study and after six months of treatment.

**Table 1:** Comparison of anthropometric parameters between female (n = 34) and male (n = 37) participants, before initiating treatment, and after six months of metformin monotherapy with lifestyle changes BMI = body mass index; WHR = waist-to-hip ratio; WHtR = waist-to-height ratio

Gender		Age (y)	Weight (kg)	Height (cm)	BMI (kg/m^2^)	Waist (cm)	Hips (cm)	WHR	W Ht R
Before initiating treatment									
Female	Mean ± SD	44.24 ± 4.64	62.71 ±10.41	149 ± 6.5	28.08 ± 4.10	94.07 ± 7.56	102.32 ± 10.45	0.92 ± 0.061	0.632 ± 0.063
Male	Mean ± SD	42.97 ± 6.53	70.11 ± 13.7	159 ± 11.5	27.64 ± 3.42	94.55 ± 7.28	93.77 ± 8.82	1.01 ± 0.04	0.60 ± 0.061
	p-Value	0.5	<0.001	<0.01	0.062	0.789	<0.001	<0.001	0.027
After six months of metformin monotherapy and lifestyle changes									
Female	Mean ± SD		60.21 ± 8.58		27.05 ± 3.71	88.33 ± 8.46	99.44 ± 9.09	0.89 ± 0.05	0.59 ± 0.06
Male	Mean ± SD		67.68 ± 13.28		26.70 ± 3.39	89.15 ± 7.29	92.42 ± 8.34	0.97 ± 0.07	0.56 ± 0.05
	p-Value		0.007		0.68	0.66	0.001	< 0.0001	0.037

The age of the female and male participants at the time of enrolment showed no significant difference. The average weight of the female participants (62.71 kg) was significantly less than the average weight of the male participants (70.11 kg). The average BMI showed no significant difference between the female (28.08 kg/m^2^) and male (27.64 kg/m^2^) subjects. While the waist circumference showed no significant difference between the females (mean 94.07 cm) and males (mean 94.55 cm), the average hip circumference was greater in females (102.32 cm) compared to males (93.77 cm), with p-values less than 0.001. The WHR was significantly higher in males, and WHtR was significantly higher in females.

After six months of treatment, both the female and male subjects lost weight, but the comparison of the BMI values and waist circumference between the female and male subjects at the end of six months of treatment showed no significant difference. The differences in hip circumferences, WHR, and WHtR continued to differ significantly between the female and male subjects.

Changes in anthropometric parameters after six months of treatment, for female and male subjects, are summarized separately in Table 2.

**Table 2:** Comparison of anthropometric parameters before initiating treatment and after six months of metformin monotherapy and lifestyle changes, in the female and male participants BMI = body mass index; WHR = waist-to-hip ratio; WHtR = waist-to-height ratio

Condition		Age (y)	Weight (kg)	Height (cm)	BMI (kg/m^2^)	Waist (cm)	Hips (cm)	WHR	W Ht R
Female participants (n = 34)									
Before treatment	Mean ± SD	44.24 ± 4.64	62.71 ±10.41	149 ± 6.5	28.08 ± 4.10	94.07 ± 7.56	102.32 ± 10.45	0.92 ± 0.061	0.632 ± 0.063
After treatment	Mean ± SD		60.21 ± 8.58		27.05 ± 3.71	88.33 ± 8.46	99.44 ± 9.09	0.89 ± 0.05	0.59 ± 0.06
	p-value		0.03		0.53	0.39	0.01	<0.001	0.01
Male participants (n = 37)									
Before treatment	Mean ± SD	42.97 ± 6.53	70.11 ± 13.7	159 ± 11.5	27.64 ± 3.42	94.55 ± 7.28	93.77 ± 8.82	1.01 ± 0.04	0.60 ± 0.061
After treatment	Mean ± SD		67.68 ± 13.28		26.70 ± 3.39	89.15 ± 7.29	92.42 ± 8.34	0.97 ± 0.07	0.56 ± 0.05
	p-value		0.44		0.24	0.002	0.50	0.008	0.01

No significant changes were observed in the BMI and waist circumference in the case of female subjects. However, changes in the hip circumference, WHR, and WHtR were statistically significant. In the case of male subjects, changes in the BMI and hip circumference were not significant, but changes in the waist circumference, WHR, and WHtR were statistically significant (p-value < 0.01).

Changes in the heart rate and blood pressure are summarized in Table 3. 

**Table 3:** Comparison of heart rate and blood pressure before initiating treatment and after six months of metformin monotherapy and lifestyle changes, in the female and male participants RHR = resting heart rate; SBP = systolic blood pressure; DBP = diastolic blood pressure

Condition		RHR (per min)	SBP (mm of Hg)	DBP (mm of Hg)
Female participants (n = 34)				
Before treatment	Mean ± SD	79.06 ± 4.65	128.71 ± 4.82	83.35 ± 2.82
After treatment	Mean ± SD	78.06 ± 4.60	125.88 ± 3.84	81.88 ±2.080
	p-value	0.376	0.009	0.017
Male participants (n = 37)				
Before treatment	Mean ± SD	78.81 ± 5.82	130.22 ± 3.32	84.43 ± 2.87
After treatment	Mean ± SD	75.19 ± 5.72	126.32 ± 2.52	82.34 ± 3.65
	p-value	0.231	<0.0001	0.009

Statistically significant improvements in blood pressure were seen in both the female and male subjects after six months of treatment (Table 3).

Table 4 compares the plasma glucose, percent HbA1c, and lipid profiles of the female and male subjects before initiating the treatment and after six months of treatment.

**Table 4:** Comparison of plasma glucose, HbA1c, and lipid profiles, before initiating treatment and after six months of metformin monotherapy and lifestyle changes

Condition		Fasting glucose (mg/dL)	2h PP glucose (mg/dL)	HbA1c (%)	Total serum cholesterol (mg/dL)	Serum triglyceride (mg/dL)	HDL-cholesterol (mg/dL)	LDL-cholesterol (mg/dL)
Before treatment	Mean ± SD	145.55 ± 27.40	207.24 ± 47.36	6.99 ± 0.70	177.80 ± 37.89	163.06 ± 27.08	42.69 ± 11.08	116.86 ± 50.4
After treatment	Mean ± SD	132.1 ± 26.66	168.68 ± 35.24	6.36 ± 0.51	167.41 ± 34.12	157.93 ± 31.61	44.87 ± 10.81	104.88 ± 48.64
	p-value	0.003	<0.0001	<0.0001	0.088	0.301	0.237	0.152

Small, statistically significant improvements in fasting and two-hour postprandial blood glucose and % HbA1c were observed after six months of treatment. Although the lipid profile showed improvement after treatment (decrease in total cholesterol, triglycerides, and LDL, with an increase in HDL-cholesterol), these changes were not statistically significant. 

The participants were questioned regarding the adherence to treatment at the end of six months of metformin monotherapy with lifestyle changes. Metformin monotherapy was followed by all participants. Nausea, diarrhea, occasional mild headache, or weakness in the first two to three weeks of treatment were reported by 25 females and 31 males. However, these problems abated spontaneously with continued treatment. Lifestyle recommendations were followed by all male subjects and only three female subjects. Table 5 summarizes the problems reported by the female subjects. Most participants belonged to the middle class and had one to three earning members in family sizes ranging from five to 13 members.

**Table 5:** Details of female participants following or not following lifestyle changes

Recommended lifestyle changes followed	Profession	Number	Reasons for non-adherence/difficulties in adherence
Yes	Homemakers	0	No time to prepare different types of meals. No time to exercise regularly.
	Working outside home	3	
No	Homemakers	17	Objections by other members of the joint family regarding cooking "special meals" for the non-earning housewife. Family members refusing healthy food due to eating habits. No time to prepare different meals. Objections by family members regarding the wastage of leftovers. Difficulty in finding time or space to exercise. No parks in the neighborhood for daily walks. Joining a gym for regular exercise is too expensive.
	Working outside home	14	No time to prepare different meals. No time to exercise regularly.

The three women who had been able to follow the lifestyle recommendations were earning members of the family and had husbands with type 2 diabetes (2) and hypertension (2). Seventeen women (all homemakers) reported various problems due to which they had been unable to follow the lifestyle recommendations. Fourteen women who were employed had been unable to follow lifestyle recommendations due to a lack of time.

## Discussion

The waist circumferences showed no statistically significant differences between the two genders, while the WHtR was significantly higher in females, indicating greater central adiposity in females (Table 1). Hip circumference was greater in females, leading to a significantly lower WHR. These differences persisted after metformin treatment, as the WHR and WHtR improved in both genders.

In the case of women, the treatment caused small but significant weight loss and a decrease in hip circumference. The reduction in the BMI and waist circumference was not significant (Table 2). In the case of male participants, a small but significant decrease in the waist circumference was observed. The six months treatment caused a small but statistically significant reduction in the WHR and WHtR. Since 31 out of the 34 female participants had been unable to follow the lifestyle changes, the improvement in the anthropometric parameters in females can be attributed to metformin therapy. DeFronzo and Goodman (1995) had reported weight loss caused by metformin [14], which was later confirmed by larger studies, e.g., the BIGPRO study group with metformin 850 mg BID treatment of one-year duration, which found a 2 kg weight loss (P < 0.06) [15]. Weight loss caused by metformin is probably due to different mechanisms. Increased lactate production due to the inhibition of complex I of the electron transport chain causes acidosis with anorexia and protein malnutrition. Metformin has been shown to increase the secretion of GLP-1 and PYY, which are anorexic and may decrease the production of orexigenic peptide NPY, altering the food-reward relationships. It decreases hypothalamic leptin resistance [16]. In the gut, metformin suppresses appetite by causing nausea, dysgeusia, and alteration of gut microbiota.

Metformin has not been recommended for weight loss by the FDA due to its modest and inconsistent effects. However, in our study, the effect was consistent and occurred within six months of treatment.

The differences in the mean heart rate, systolic blood pressure, and diastolic blood pressure between females (79.06 per minute, 128.71 mm of Hg, and 83.35 mm of Hg, respectively) and males (78.81 per min, 130.22 mm of Hg, and 84.43 mm of Hg, respectively) was not statistically significant (p-values > 0.05). Similarly, after six months of treatment, the differences in the mean heart rate and blood pressure between the female and male subjects were not statistically significant.

Although the mean heart rate of both the female and male participants decreased after six months of treatment, the change was not statistically significant (Table 3). The decrease in systolic and diastolic blood pressure after therapy was significant in both genders. The change in blood pressure in the case of the female subjects was due to metformin monotherapy alone (only three female participants had been able to follow the lifestyle changes), while in the case of the male participants, it was due to both lifestyle changes and metformin. Zhou et al. [17] have reported a decrease in systolic blood pressure, while Junior et al. [18] have reported no effect of metformin on blood pressure. In the above studies, the patients were non-diabetic. In our study, a decrease in both systolic and diastolic blood pressure was observed after six months of treatment. Since the female group had not followed lifestyle changes, the changes in blood pressure could be due to metformin alone. Further research is needed using placebo trials.

Metformin monotherapy with (males) and without (females) lifestyle changes showed significant differences in plasma glucose (fasting and two-hour postprandial) and percent HbA1c (Table 4). Data from only the 31 female participants who did not follow lifestyle changes is presented in this table. Mean serum cholesterol, LDL-cholesterol, and triglyceride levels decreased after six months of therapy, while serum HDL-cholesterol increased in both female and male patients, but this improvement was not statistically significant, probably due to the large SD values and small sample size. Meta-analyses of RCTs by Gillani et al. and Weng et al. [11,19] showed that metformin improves lipid profiles in type 2 diabetics and non-diabetics. Differences in our studies could be due to the small sample size or short duration of the study. They could also be due to genetic differences in the Indian population.

This study showed the difficulties encountered by Indian women in following lifestyle changes recommended by the physician (Table 5). It also indicates that the routine diet in many Indian families is not healthy, which should be taken by persons at risk of developing T2DM. Due to financial constraints or other reasons, many homemakers have to compromise with their health. Public awareness is required regarding these issues, such that a healthy diet should be preferred by the entire family and not just the patient and that homemakers may not be the breadwinners but are the bread makers and back bones of the family. Healthy cooking methods that are easy, less time-consuming, and do not compromise taste can be developed and taught in schools and through social media. 

A large clinical trial conducted on 3234 non-diabetics using 850 mg metformin BD showed reduced incidence of diabetes in persons at high risk [20]. Similar trials on South Asian and Indian populations could help establish the efficacy of metformin in these people.

Limitations of the study

This pilot study had 71 participants (34 female). This small sample size was further compromised as 31 female participants did not follow the lifestyle changes. All participants were from the same ethnic background and resided in neighboring areas, compromising diversity. The time duration of the study was short (six months) and showed only short-term changes in all parameters.

## Conclusions

Metformin monotherapy (500 mg OD) with (males) or without (females) lifestyle changes over a period of six months showed significant improvement in the WHR, WHtR, blood pressure, plasma glucose, and percent HbA1c in Indian patients with newly diagnosed T2DM. Improvement in the lipid profile was not statistically significant. The female participants reported difficulties in following lifestyle changes recommended by the physician. The side effects of metformin treatment were mild and disappeared in two to three weeks.
